# Fear of cancer recurrence in patients with multiple myeloma: Prevalence and predictors based on a family model analysis

**DOI:** 10.1002/pon.5546

**Published:** 2020-10-01

**Authors:** Xiaochun Hu, Weida Wang, Ye Wang, Ke Liu

**Affiliations:** ^1^ Department of Hematologic Oncology Sun Yat‐sen University Cancer Center State Key Laboratory of Oncology in South China Collaborative Innovation Center for Cancer Medicine Guangzhou Guangdong China; ^2^ School of Nursing Sun Yat‐sen University Guangzhou Guangdong China

**Keywords:** cancer, family factors, fear of cancer recurrence, multiple myeloma, oncology, psycho‐oncology

## Abstract

**Objective:**

Fear of cancer recurrence (FCR) is a common psychosocial sequela among cancer survivors, but data on patients with multiple myeloma are scarce. This study calculated the prevalence of FCR and identified family and social factors that predict FCR in the study population.

**Methods:**

We recruited 127 myeloma patients and their partners to participate in a cross‐sectional survey from a regional tertiary cancer centre in China. The questionnaires included items on demographic characteristics and from the fear of disease progression simplified scale, family hardiness index and Social Support Scale. Univariate and multivariate regression was used to identify predictors of FCR.

**Results:**

Of the participants, 56.4% patients reported high‐level FCR, which was similar to the partner‐reported proportion. The partners' FCR was positively associated with the patients' FCR, while family hardiness and social support were statistically significant, negative predictors.

**Conclusions:**

Interventions to mitigate partners' FCR and improve family hardiness and social support may help with the psychological adjustment and well‐being of myeloma patients.

## 1 INTRODUCTION

Multiple myeloma is the second most common haematological malignancy and is characterised by extensive lytic bone lesions, renal impairment, anaemia, recurrent infections and hypercalcaemia. Despite emerging novel agents and approaches, it remains incurable. The median progression‐free survival in China is estimated to be as long as 16 months, which is comparable to data in other countries.^1^ However, the median overall survival of myeloma patients is about 7–10 years, which implies that most patients will eventually experience several relapses and/or progressions.

Fear of cancer recurrence (FCR), defined as ‘fear, worry or concern relating to the possibility that cancer will come back or progress’,^2^ is the most frequent distressing symptom among cancer patients and their partners.^3^ Simard and colleagues^4^ found that 39%–97% of cancer patients may have a degree of FCR. FCR is considered one of the cancer survivors' greatest concerns^5^ and is associated with post‐traumatic stress disorder, inferior prognosis and lower well‐being and global quality of life. Cancer impacts patients and their caregivers and family members negatively.^6^, ^7^ Mellon and colleagues proposed a family based model to analyse predicting factors of FCR in patients and their family caregivers.^8^ According to Mellon's model, both individual and dyadic factors were associated with FCR. Personal factors, such as a partner's age, individual and concurrent family stressors and a patient's appraisal of the illness, could predict individuals' degree of FCR. A patient's FCR was found to be interdependent with that of the caregiver. The model is critical for addressing potential factors influencing patients' and their family members' FCR. Using this family model as a conceptual framework, we conducted the present study with the aims of (1) investigating myeloma survivors' residual FCR issues after completing conventional treatment and (2) uncovering relationships between demographic data, partners' FCR, family hardiness, social support and the FCR of myeloma patients in China.

## 2 METHODS

### 2.1 Participants

Convenience sampling was used to recruit participants. The inclusion criteria for patients were: (1) previously diagnosed with multiple myeloma according to the Multiple Myeloma Diagnosis and Treatment Guidelines^9^; (2) awareness of disease diagnosis and condition; (3) completed conventional treatment, in the maintenance phase, currently in remission or disease control without a history of recurrence; (4) able to understand the contents of the questionnaires; able to speak and read in Chinese Mandarin or Cantonese and (5) provided consent to participate. We excluded patients with concurrent major physical or mental health problems (e.g., dementia, acute myocardial infarction). For patients who met the inclusion criteria, further inclusion reviews for their spouses included: (1) married and living with the patient, (2) willing to participate in this study, (3) able to understand the contents of the questionnaires and (4) able to speak and read in Chinese Mandarin or Cantonese. During the study period from October 2018 to September 2019, 127 pairs of myeloma patients and partners were enrolled at Sun Yat‐sen University Cancer Center. All patients were married.

### 2.2 Measures

We took a dyadic approach to investigate patients and their spouses. All patients completed four in‐person questionnaires to gather personal information and assess FCR, family hardiness and social support. Their spouses completed two questionnaires to gather personal information and assess FCR. After completing the questionnaires, in‐person interviews were conducted with the patients and their spouses separately. Missing items were confirmed with the participants, using questions, such as ‘Did you have any problems in answering this question?’ and ‘What do you think about this question?’. The investigators encouraged the participants to provide details and to clarify their statements. Demographic data, including age, gender, education level, residence, occupation, income level, quantity of children, religion and healthcare coverage, were assessed via participant self‐reports. ‘Need for psychological consultation’ was evaluated using questions such as ‘Did you ever consider of seeking advice from psychological consultants?’ and ‘How often do you think about this question?’. The investigators collected clinical data, including myeloma stage, duration since diagnosis and comorbidity.

FCR was measured with the Fear of Progression Questionnaire short form (FoP‐Q‐sf), which is a 12‐item, bidimensional (physical health and social‐familial) scale^10^ that was simplified from the original 43‐item version.^11^ The items are scored on a 5‐point Likert scale ranging from 1 (‘never’) to 5 (‘always’). The potential total score ranged from 12 to 60. A higher score indicates a higher level of FCR. According to a previous study by Herschbach et al.,^12^ the cut‐off for dysfunctional FCR is ≥ 34. The Chinese version of the questionnaire demonstrated high internal and test‐retest reliability and high consistency in Singapore.^13^

The partners' FCR was measured with the FoP‐Q‐sf/P, the only instrument available to evaluate partners' FCR at present. It was developed by Zimmermann^14^ and is based on the FoP‐Q‐sf; thus, it has the same cut‐off value.

Family resilience was assessed using the Family Hardiness Index (FHI), a 20‐item, tridimensional (commitment, challenge and control) scale. It was developed by McCubbin in 1986 to evaluate the internal strengths of family members.^15^ The FHI totals all the responses to every item and has a possible score range of 0–60. There is no cut‐off value to avoid discriminating against participants. A higher score indicates a higher level of family hardiness.

Social Support Rating Scale (SSRS) assesses the strength of social support. This 10‐item, tridimensional scale was developed by Xiao^16^ and has been widely used in China. For the classification of low, moderate, good and high levels of social support, the cut‐off values are 26, 36 and 46, respectively.

### 2.3 Ethics

The present study was approved by the Research Ethics Committee of Sun Yat‐sen University Cancer Center (Approval Number: B2020‐054‐01) and conducted following the Declaration of Helsinki. Data collection permission was acquired from hospital administrators. Written consent was obtained before collection, and anonymity was ensured. Trained investigators performed the data collection and assistance with completing questionnaires when needed.

### 2.4 Statistics

Data analysis was conducted using SPSS version 20. Frequency, percentage, mean and standard deviation were used to describe demographic and clinic data. The mean and standard deviation were calculated to explore partners' and patients' FCR and FHI and SSRS scores. Pearson correlations were used to measure the relationship between the variables and FCR. We performed multivariate regression analysis on the variables that were significantly associated with FCR (*p* < 0.05).

## 3 RESULTS

### 3.1 Demographics, clinical characteristics and FCR

The ages of the 127 patients ranged from 28 to 80 years, with a mean age of 58.09 years (SD = 9.52). Of the patients, 61.4% (*n* = 54) were male, and 25.2% (*n* = 32) had graduated college or above. Of the patients, 41.8% (*n* = 53) lived in a village and worked in agriculture (40.2%, *n* = 51). The majority (87.2%, *n* = 111) of the patients were retired or on sick leave, and 93.7% declared no religion. The ages of the 127 corresponding partners ranged from 27 to 80 years, with a mean age of 57.71 years (SD = 9.96). Of the partners, 12.6% (*n* = 16) had graduated from college or above. About 43.3% (*n* = 55) of the patients had a monthly household income per person below 450 USD, the average monthly income of the population in Guangdong, China. The descriptive statistics and correlations between demographic data, clinical characteristics and FCR are presented in Table 1. Age of participant, time since diagnosis, comorbidity, household income and self‐reported need for psychological consultation were statistically significantly associated with participants· FCR levels (*p* < 0.05; Table 1).

**TABLE 1:** Demographic characteristics and comparisons of FCR among subgroups

Parameter	*N* = 127	FCR (mean ± SD)	*F*/*t*	*p*
Age (years)			−2.332	0.021
<60	73	36.99 ± 11.51	‐	‐
≥60	54	32.43 ± 9.995	‐	‐
Gender			−1.415	0.16
Male	78	33.95 ± 11.12	‐	‐
Female	49	36.80 ± 10.91	‐	‐
Education			3.229	0.055
Elementary school and below	42	35.33 ± 10.53	‐	‐
Junior school	33	38.76 ± 10.94	‐	‐
High school	20	29.30 ± 10.29	‐	‐
College and above	32	34.44 ± 11.31	‐	‐
Residence			0.29	0.749
County and above	47	34.79 ± 10.83	‐	‐
Town	27	36.48 ± 12.33	‐	‐
Village	53	34.55 ± 10.80	‐	‐
Religion			0.835	0.405
w/	8	31.88 ± 10.15	‐	‐
w/o	119	35.26 ± 11.15	‐	‐
Current working status				1.399
Working	16	31.44 ± 11.30	‐	‐
Retired/sick leave	111	35.57 ± 11.01	‐	‐
Occupation			1.918	0.13
Office clerk/worker	59	35.49 ± 10.54	‐	‐
Agricultural worker	51	34.67 ± 10.42	‐	‐
Self‐employed	7	27.14 ± 16.17	‐	‐
Freelance	10	39.90 ± 12.23	‐	‐
Time since diagnosis (years)			3.262	0.024
<1	118	36.71 ± 10.95	‐	‐
1∼	55	34.87 ± 13.08	‐	‐
2∼	24	26.10 ± 5.82	‐	‐
≥3	54	35.47 ± 9.34	‐	‐
Stage of disease			2.054	0.133
Ⅰ	66	33.15 ± 10.81	‐	‐
II	34	36.91 ± 11.45	‐	‐
Ⅲ	27	37.33 ± 10.85	‐	‐
Comorbidity			2.146	0.034
w/	49	37.67 ± 11.07	‐	‐
w/o	78	33.40 ± 10.84	‐	‐
Therapeutic modality			0.201	0.841
Chemotherapy	103	34.95 ± 11.22	‐	‐
Multidisciplinary	24	35.46 ± 10.69	‐	‐
Household monthly income per person (USD)			2.479	0.015
<450	55	37.78 ± 11.32	‐	‐
≥450	72	32.96 ± 10.51	‐	‐
Caregiver			0.763	0.468
Spouse	78	34.95 ± 11.67	‐	‐
Sibling	42	36.00 ± 9.91	‐	‐
Other	7	30.43 ± 11.36	‐	‐
Health care coverage			1.267	0.289
At state expense	6	26.67 ± 8.89	‐	‐
Medical insurance	58	35.90 ± 11.35	‐	‐
NCMS	50	35.12 ± 10.55	‐	‐
Other	13	34.85 ± 12.34	‐	‐
Number of children			1.665	0.193
1	36	33.97 ± 10.64	‐	‐
2	43	37.53 ± 10.38	‐	‐
≥3	48	33.63 ± 11.84	‐	‐
Need for psychological consultation			9.927	<0.001
Never	64	31.13 ± 9.77	‐	‐
Little	35	35.91 ± 10.48	‐	‐
Sometimes	17	38.41 ± 10.14	‐	‐
Often	6	46.33 ± 5.75	‐	‐
Always	5	54.20 ± 5.50	‐	‐
Partner demographics age (years)			2.136	0.055
<60	71	36.89 ± 11.62	‐	‐
≥60	56	32.71 ± 9.99	‐	‐
Education			2.726	0.053
Elementary school and below	39	36.28 ± 10.35	‐	‐
Junior school	40	37.15 ± 11.71	‐	‐
High school	32	30.34 ± 10.99	‐	‐
College and above	16	36.19 ± 9.488	‐	‐
Religion			1.89	0.061
w/o	120	35.49 ± 11.05	‐	‐
w/	7	27.43 ± 9.36	‐	‐
Current working status			0.02	0.517
Working	54	33.72 ± 10.82	‐	‐
Retired/sick leave	73	36.03 ± 11.25	‐	‐
Occupation			1.54	0.208
Office clerk/worker	50	33.78 ± 9.87	‐	‐
Agricultural worker	53	35.09 ± 10.90	‐	‐
Self‐employed	8	32.38 ± 14.05	‐	‐
Freelance	16	40.19 ± 13.14	‐	‐

Abbreviations: FCR, fear of cancer recurrence; NCMS, new rural cooperative medical system.

### 3.2 All participants' FCR and patients' FHI and SSRS scores

Overall, 56.4% (*n* = 72) of patients and 63% (*n* = 80) of partners reported experiencing high‐level FCR. The average FHI score of the 127 patients was 57.65 (SD = 7.73). Among the three subscales of FHI, the average commitment score was 27.19 (SD = 4.18), with 16.10 (SD = 3.59) in the control subscale, and 14.37 (SD = 2.19) in the challenge subscale. The average SSRS score of the 127 patients was 40.68 (SD = 7.98), with 2 (1.6%) classified as low (19–25), 24 (18.9%) classified as moderate (26–35), 58 (45.7%) classified as good (36–45) and 43 (33.9%) classified as high (>45). The descriptive analyses of FCR, FHI and SSRS scores are presented in Table 2.

**TABLE 2:** Patient and partner FCR and patients' FHI and SSRS scores (*n* = 127)

Variable	Possible range	Range of score	mean ± SD
Patient FCR	12–60	12–60	35.05 ± 11.09
Partner FCR	12–60	12–60	35.57 ± 10.11
FHI	20–80	39–77	57.65 ± 7.73
SSRS	14–66	19–59	40.68 ± 7.98

Abbreviations: FCR, fear of cancer recurrence; FHI, family hardiness index; SSRS, Social Support Rating Scale.

### 3.3 Correlations between family factors and participants' FCR

Pearson correlation was computed to evaluate whether there were relationships between patients' FCR, FHI and SSRS scores and partners' FCR. The results indicated that patients' FCR was highly correlated with that of their partners, while higher family hardiness and social support were significantly negatively related to the patients' FCR (see Table 3). FHI was significantly associated with SSRS. Partners' FCR was independent of FHI and SSRS.

**TABLE 3:** Relationships between partner FCR, FHI, and SSRS and patient FCR (*r* ^2^, *n* = 127)

	Patient's FCR	Partner's FCR	FHI	SSRS
Patient's FCR	1	0.614**	−0.267**	−0.287**
Partner's FCR	0.614**	1	−0.137	−0.083
FHI	−0.267**	−0.137	1	0.332**
SSRS	−0.287**	−0.083	0.332**	1

Abbreviations: FCR, fear of cancer recurrence; FHI, family hardiness index; SSRS, Social Support Rating Scale.

**p < 0.01.

### 3.4 Predictive and protective factors of participants' FCR

Multivariate linear regression analysis was conducted to identify the variance in FCR accounted for by demographic, clinical and family factors and is presented in Tables 1, 2, 3. Based on the significant results between candidate predictors and FCR, these variables were validated in multivariate linear regression (see Table 4). Thus, age, occupation, time since diagnosis, comorbidity, monthly household income per person, self‐reported need for psychological consultation, one's partner's FCR and the FHI and SSRS scores entered the final model. Age, comorbidity, lower income, self‐reported need for psychological consultation and one's partner's FCR were positive predictors of a patient's FCR. Higher FHI and SSRS scores were negative predictors, or protectors, of a patient's FCR. These variables accounted for 58% of the variance (*p* < 0.01). The analysis was found to be statistically significant F (9, 117) = 18.289, *p* < 0.01.

**TABLE 4:** Independent variable assignment and regression analysis of factors associated with patient FCR

Variable	Assignment
Time since diagnosis	1 =<1year; 2 = 1–2years; 3 = 2–3years; 4 = ≥3years
Comorbidity	0 = without; 1 = with
Need for psychological consultation	1 = never; 2 = little; 3 = sometimes; 4 = often; 5 = always
Household monthly income (USD)	1 =<450; 2 = ≥450
Occupation	1 = office clerk/worker; 2 = agricultural worker; 3 = self‐employed; 4 = freelance
Age	Original value
FHI	Original value
SSRS	Original value
Partner FCR	Original value

Variable	*B*	*Sx*	*T*	*p*
Constant	34.894	7.063	4.941	0.000
Age	−3.171	1.397	2.270	0.025
Occupation	−0.764	0.797	−0.958	0.340
Time since diagnosis	−1.036	0.568	−1.823	0.071
Comorbidity	3.319	1.379	2.407	0.018
Household monthly income per person	−2.862	1.374	−2.082	0.040
Need for psychological consultation	3.015	0.664	4.539	<0.001
Partner's FCR	0.471	0.072	6.514	<0.001
FHI	−0.200	0.095	−2.102	0.038
SSRS	−0.220	0.092	−2.392	0.018
*F* = 18.289, *p* < 0.01, *R* ^*2*^ = 0.585, *R*_*ad*_ = 0.553				

Abbreviations: FCR, fear of cancer recurrence; FHI, family hardiness index; SSRS, Social Support Rating Scale.

## 4 DISCUSSION

Using Mellon's family based FCR model as a conceptual framework, this study indicated that FCR occurs in both myeloma patients and their spouses. We described the prevalence of high‐level FCR in myeloma patients and explored factors related to FCR, including demographic, physical and psychosocial factors. Among the myeloma survivors who completed conventional treatments, 56% had high‐level FCR. Physical, stress, family and social factors can impact patients' FCR.

Addressing the issue of whether FCR occurs differently according to cancer type, it has been suggested that having skin, colon or haematological cancer might predict FCR.^17^ The occurrence and intensity of FCR are greatly impacted by perceived controllability, a consequence of recurrence, treatment modality and the time‐course of cancer.^18^ Multiple myeloma, as an incurable haematological malignancy,^19^ has several characteristics that can cause fear. A relatively longer survival period than other advanced stage cancers can result in a persistent confrontation with potential progression. Asymptomatic relapse or progression before symptomatic recurrence can mean recurrence might be invisible. Bone pain, which is the most common, obvious symptom, can contribute to depression and anxiety. Novel agents that compose the standard regimen are still expensive for the majority of Chinese patients, and recurrence might mean a continually updated and/or extended use of novel agents. According to a survey on patients' lived experience of myeloma,^20^ participants had a distinctive experience in living with this form of cancer, and their fears need to be addressed. In our cohort of 127 myeloma patients, the mean FCR score was 35.05 (SD = 11.09), with 56.4% classified as high‐level or maladaptive FCR. In line with other studies conducted in China on participants with breast cancer^21^ and gynaecologic cancer,^22^ FCR was highly prevalent in myeloma survivors. FCR, to a certain degree, is a natural concern due to a real threat, and it may even be adaptive. Moderate FCR may increase patients' compliance and vigilance. However, excessive FCR can become clinically relevant.^23^ Among haematological malignancies, FCR has an intermediary role in the relationship of bodily symptoms and quality of life.^24^ Severe FCR has a negative impact on survival in the lymphoma population.^25^ The present study highlighted the differential need of the myeloma population and provided a wealth of data to inform the planning and implementation of targeted interventions.

Concerns over recurrence may also affect spouses and caregivers. This study corroborated the findings of previous studies that FCR is not restricted to cancer survivors but also affects partners.^8^ For instance, Marieke and colleagues^26^ and Cohee and colleagues^27^ reported equal mean FCR scores between survivors and partners, regardless of gender. In our cohort of 127 partners, the mean FCR score was 35.57 (SD = 10.11), slightly higher than that of patients. Univariate and multivariate analysis indicated that there was a positive correlation between partner's and patient's FCR. Overall, this study provides early evidence that partners' FCR is related to that of patients. We do not know whether the levels are causally linked or concurrent. Nevertheless, this study indicates interventions aimed at patients alone might be insufficient. Family factors deserve more attention.

We identified several demographic and clinical factors associated with patients' FCR, including age, comorbidity and monthly income. In accordance with previous studies,^28^ age was significantly and negatively related to FCR. Comorbidities might be related to persistent apprehension, uncertainty and distress, which might eventually amplify FCR. Financial factors are undeniably crucial to the accessibility of therapies after recurrence. According to a study in survivors of haematopoietic stem cell transplantation,^29^ financial stressors, insurance stressors and employment were associated with poorer health‐related quality of life. In our study, financial factor was associated with FCR. This might indicate financial factor could impact profoundly in multiple aspects of physical and mental well‐beings of cancer patients.

This study failed to find a statistically significant impact of time since diagnosis on FCR. We found that patients within the first year of diagnosis reported higher levels of FCR than those who had been diagnosed 1–2 years earlier. However, in patients 3 years after diagnosis, FCR levels were higher than those who had been diagnosed 1–2 years earlier, but similar to those who were within the first year of diagnosis. We assume this phenomenon is attributed to a patient's life transition and the time‐course of myeloma. Unlike other cancers, myeloma will inevitably relapse, and most relapses occur 3 years after diagnosis. A 2013 systematic review of patients with other malignancies indicated that FCR tends to remain stable over time,^4^ but a later study that was conducted longitudinally in prostate cancer patients^30^ showed a decrease in FCR over time suggesting that time post‐treatment may have ameliorated patients' FCR. However, in the case of multiple myeloma, the impact of the disease course may be different to other cancers. Multiple myeloma typically has a long disease course and multiple recurrences are expected. A longitudinal study would provide more information about FCR in this population. The self‐reported need for psychological consultation might not predict FCR; instead, it may represent awareness of FCR and the unmet need for psychological rehabilitation in myeloma survivors. This area of research requires further investigation.

This study indicates that FHI scores are negatively related to FCR. The FHI was developed to measure family stress resistance and adaption resources, which refer to a family's ability to work together, confidence in handling problems, approach and attitude to new experiences, and sense of being in control of family life.^31^ The more resilient a patient's family is, the less fear the patient feels toward cancer recurrence. Evidence of the alleviation of family hardiness on the stress response of family members has been supported in previous studies.^32^, ^33^ Walsh^34^ suggested that the capacity to handle problems as a family is crucial to facing a crisis. A family's internal strengths and resilience augment and contribute to the entire family's appraisal and sense of meaning. Family hardiness serves as an important resource in predicting a patient's appraisal of illness.^35^, ^36^ The entire family's maintenance of a positive, optimistic attitude might boost a patient's confidence and, thus, mitigate his FCR.

The second resource, social support, had a significant and direct negative effect on patients' FCR. Other studies have also shown that patients who report more social support have a lower level of FCR.^37^ Support received from family members, friends and health professionals might reduce patients' stress, improve their confidence and compliance, and help their rehabilitation.^38^ As an available external resource,^39^, ^40^ social support plays an important role in keeping family functions in balance.

### 4.1 Study limitations

Despite the supportive findings of this study, several limitations need to be acknowledged. First, this study uses a cross‐sectional design; a longitudinal approach might provide a stronger determination of FCR changes over time. Second, although the FoP‐Q‐sf is a validated measure with excellent internal consistency, its clinical cut‐off value has not been established so far. We chose an empirical cut‐off value; however, a ‘maladaptive’ level of FCR is difficult to diagnose as ‘clinical’ without typical pathological manifestations. A third limitation is the homogeneity of the marital status of the enrolled participants. All were married and lived with family members. Live‐in partnerships are in the minority and usually undeclared due to the relatively conservative social circumstances in China. Moreover, non‐binding relationships were difficult to define. We aimed to examine family factors in a patient's FCR; therefore, to avoid the influences of other partnerships on family factors, we restricted the inclusion criteria to the most restrictive constant—married. In turn, the results should be interpreted conservatively. Survivors who are divorced, widowed, or single might be more vulnerable to FCR due to their lack of family and social support, and they may need more attention. This sampling bias can be resolved in future research.

### 4.2 Clinical implications

Overall, this study has several clinical implications. First, the present study focused on a specific psychosocial issue in the Chinese population. There may be differences from people from other countries in terms of culture, family value, and religious beliefs. The present study described the prevalence of FCR in a Chinese population cohort. FCR is a common issue in China as it is among people in other countries. However, the severity of FCR in Chinese patients might be different, since family elements play an important role in ameliorating FCR and Chinese, especially Cantonese people, usually have extended family and extensive relationship networks, which might strengthen family hardiness and social support. To clarify these effects, inclusion of a detailed measure of family and social relationships ought to be included in any further investigation. Second, we conducted a dyadic approach to investigate patients and their spouses. Our finding shed light on the close link between patient's FCR and the spouse's. Third, patients with multiple myeloma are vulnerable to FCR. Addressing their fears, and exploring relevant co‐variates are meaningful to mitigate FCR in this population.

## 5 CONCLUSIONS

Most multiple myeloma survivors completing conventional treatments report fear of recurrence. Several demographic and medical factors are helpful in predicting FCR. To mitigate FCR, partner factors, family hardiness and social support should be addressed during rehabilitation and follow‐up care.

## CONFLICT OF INTEREST

The authors have no conflict of interest to declare.

## Data Availability

The data that support the findings of this study are available from the corresponding author upon reasonable request.

## References

[1] Mateos MV , Hernandez JM , Hernandez MT , et al. Bortezomib plus melphalan and prednisone in elderly untreated patients with multiple myeloma: update time to events results and prognostic factors for time to progression. Haematologica. 2008;93(4):560‐565. 18322252 10.3324/haematol.12106

[2] Lebel S , Ozakinci G , Humphris G , et al. From normal response to clinical problem: definition and clinical features of fear of cancer recurrence. Support Care Cancer. 2016;24(8):3265‐3268. 27169703 10.1007/s00520-016-3272-5

[3] Zahlis EH , Shands ME . Breast cancer demands of the illness on the patients partner. J Psychosoc Oncol. 1991;9(1):75‐93.

[4] Simard S , Thewes B , Humphris G , et al. Fear of cancer recurrence in adult cancer survivors: a systematic review of quantitative studies. J Cancer Surviv. 2013;7(3):300‐322. 23475398 10.1007/s11764-013-0272-z

[5] Ozga M , Aghajanian C , Myers‐Virtue S , et al. A systematic review of ovarian cancer and fear of recurrence. Palliat Support Care. 2015;13(6):1771‐1780. 25728373 10.1017/S1478951515000127 PMC4995592

[6] Heydarnejad MS , Dehkordi A , Dehkordi K . Factors affecting quality of life in cancer patients undergoing chemotherapy. Afr Health Sci. 2011;11(2):266‐270. 21857860 PMC3158510

[7] Meacharoen W , Sirapongam Y , Monkong S . Factors influencing quality of life among family caregivers of patients with advanced cancer patients: a casual model. Pacific Rim Int J Nurs Res. 2013;17(4):304‐316.

[8] Mellon S , Kershaw TS , Northouse LL , Freeman‐Gibb L . A family‐based model to predict fear of recurrence for cancer survivors and their caregivers. Psychooncology. 2007;16(3):214‐223. 16906624 10.1002/pon.1074

[9] Rajkumar SV , Dimopoulos MA , Palumbo A , et al. International Myeloma Working Group updated criteria for the diagnosis of multiple myeloma. Lancet Oncol. 2014;15(12):e538‐e548. 25439696 10.1016/S1470-2045(14)70442-5

[10] Hinz A , Mehnert A , Ernst J , Herschbach P , Schulte T . Fear of progression in patients 6 months after cancer rehabilitation‐a validation study of the fear of progression questionnaire FoP‐Q‐12. Support Care Cancer. 2015;23(6):1579‐1587. 25412727 10.1007/s00520-014-2516-5

[11] Herschbach P , Berg P , Dankert A , et al. Fear of progression in chronic diseases: psychometric properties of the Fear of Progression Questionnaire. J Psychosom Res. 2005;58(6):505‐511. 16125517 10.1016/j.jpsychores.2005.02.007

[12] Herschbach P , Berg P , Waadt S , et al. Group psychotherapy of dysfunctional fear of progression in patients with chronic arthritis or cancer. Psychother Psychosom. 2010;79(1):31‐38. 10.1159/000254903. 19887889

[13] Mahendran R , Liu J , Kuparasundram S , Griva K . Validation of the English and simplified Mandarin versions of the fear of progression questionnaire ‐ short form in Chinese cancer survivors. BMC Psychol. 2020;8(1):10. 32005291 10.1186/s40359-020-0374-0 PMC6995061

[14] Zimmermann T , Herschbach P , Wessarges M , Heinrichs N . Fear of progression in partners of chronically ill patients. Behav Med. 2011;37(3):95‐104. 21895427 10.1080/08964289.2011.605399

[15] McCubbin MA , McCubbin HI , McCubbin HI . Resiliency in families: a conceptual model of family adjustment and adaptation in response to stress and crises. In: Thompson AI , McCubbin MA , eds. Family Assessment: Resiliency, Coping, and Adaptation‐Inventories for Research and Practice. Madison, WI: University of Wisconsin System; 1996:1‐64.

[16] Xiao S , Yang D , Hui S , et al. Effects of social support on physical and psychological health. Chin Ment Health J. 1987;4:183‐187.

[17] Mehnert A , Koch U , Sundermann C , Dinkel A . Predictors of fear of recurrence in patients one year after cancer rehabilitation: a prospective study. Acta Oncol. 2013;52(6):1102‐1109. 23384721 10.3109/0284186X.2013.765063

[18] Latella LE , Rogers M , Leventhal H , et al. Fear of cancer recurrence in lymphoma survivors: a descriptive study. J Psycho Oncol. 2020;38(3):251‐271. 10.1080/07347332.2019.1677840. PMC7159999 31617830

[19] Zhou Y , Zuo L , Li L , et al. Observation of effect of lenalidomide in multiple myeloma. J Qilu Nurs. 2013;19(3):5‐7.

[20] Kelly M , Dowling M . Patients’ lived experience of myeloma. Nurs Stand. 2011;25(28):38‐44. 10.7748/ns2011.03.25.28.38.c8397 21488448

[21] Zhang Y , Chen C , Cui P , et al. Description and impact factors of fear of cancer recurrence in breast cancer survivors. Chin Gen Prac. 2018;21(20):2479‐2483.

[22] Liao L , Wang Z , Hu J , et al. Description and impact factors of fear of cancer recurrence in gynecological cancer survivors completing surgery and chemotherapy. Acta Acad Med Zunyi. 2018;41(5):630‐634.

[23] Herr NR , Williams JW, Jr., Benjamin S , McDuffie J . Does this patient have generalized anxiety or panic disorder? The rational clinical examination systematic review. JAMA. 2014;312(1):78‐84. 25058220 10.1001/jama.2014.5950

[24] Esser P , Götze H , Mehnert‐Theuerkauf A , Knoop H , Kuba K . Fear of progression and its role in the relationship of cancer‐related fatigue with physical functioning and global quality of life ‐ a register‐based study among hematological cancer survivors. J Psychosom Res. 2019;127:109844. 31707130 10.1016/j.jpsychores.2019.109844

[25] Kim SJ , Kang D , Kim IR , et al. Impact of fear of cancer recurrence on survival among lymphoma patients. Psychooncology. 2020;29(2):364‐372. 10.1002/pon.5265. 31654534

[26] van de Wal M , Langenberg S , Gielissen M , Thewes B , van Oort I , Prins J . Fear of cancer recurrence: a significant concern among partners of prostate cancer survivors. Psychooncology. 2017;26(12):2079‐2085. 28317267 10.1002/pon.4423

[27] Cohee AA , Adams RN , Johns SA , et al. Long‐term fear of recurrence in young breast cancer survivors and partners. Psychooncology. 2017;26(1):22‐28. 26490953 10.1002/pon.4008 PMC4840075

[28] Chumdaeng S , Soivong P , Sethabouppha H , Chontawan R . Health problems among breast cancer survivors after completing conventional treatments: a cross‐sectional study. Nurs Health Sci. 2020;22(2):436‐444. 10.1111/nhs.12678. 31943652

[29] Hamilton JG , Wu LM , Austin JE , et al. Economic survivorship stress is associated with poor health‐related quality of life among distressed survivors of hematopoietic stem cell transplantation. Psychooncology. 2013;22(4):911‐921. 10.1002/pon.3091. 22605430 PMC3648213

[30] Wu LM , McGinty H , Amidi A , Bovbjerg K , Diefenbach MA . Longitudinal dyadic associations of fear of cancer recurrence and the impact of treatment in prostate cancer patients and their spouses. Acta Oncol. 2019;58(5):708‐714. 30741082 10.1080/0284186X.2018.1563714 PMC6534441

[31] Persson C , Benzein E , Årestedt K . Assessing family resources: validation of the Swedish version of the Family Hardiness Index. Scand J Caring Sci. 2016;30(4):845‐855. 26766613 10.1111/scs.12313

[32] Greeff AP , Vansteenwegen A , Geldhof A . Resilience in families with a child with cancer. Pediatr Hematol Oncol. 2014;31(7):670‐679. 24851714 10.3109/08880018.2014.905666

[33] Park IS , Tak YR , Lee JA . Effects of family value on family adaptation in family who has a child with cancer. J Korean Acad Child Health Nurs. 2001;7(4):494‐510.

[34] Walsh F . A family resilience framework: innovative practice applications. Fam Relat. 2002;51(2):130‐137.

[35] Bodenmann G , Revenson T . Dyadic coping and its significance for marital functioning. Psychological Association. 2005;11(7):33‐49.

[36] Mellon S , Northouse LL . Family survivorship and quality of life following a cancer diagnosis. Res Nurs Health. 2001;24(6):446‐459. 11746074 10.1002/nur.10004

[37] Cai J , Guo B , Jiang Z , et al. Description and impact factors of fear of cancer recurrence in breast cancer survivors. J Nurs. 2018;25(21):5‐8.

[38] Akechi T , Momino K , Iwata H . Brief screening of patients with distressing fear of recurrence in breast cancer survivors. Breast Cancer Res Tr. 2015;153(2):475‐476. 10.1007/s10549-015-3537-6 26261002

[39] Xiaohong L , Qi W , Zheng A , et al. Discussions on psychological mental care and social support on advance cancer patients. Chin Nurs Manag. 2018;18(3):289‐293.

[40] Yang W , Zhang L , Hou L , et al. Correlation study on social support and quality of life of male NSCLC patients with various p53 expression. Beijing Med J. 2018;40(1):1‐7.

